# Production and nutritional value of milk from Creole goats fed with different levels of sweet potato vine flour (*Ipomoea batata*)

**DOI:** 10.5455/javar.2026.m1051

**Published:** 2026-06-22

**Authors:** Fritz Trillo-Zárate, Emmanuel Alexander Sessarego, Irene Acosta, José Ruíz-Chamorro, Aníbal Rodríguez-Vargas, Juancarlos Cruz

**Affiliations:** 1 Dirección de Servicios Estratégicos Agrarios, Instituto Nacional de Innovación Agraria (INIA), Av. La Molina 12791, Lima, Perú; 2 Estación Experimental Agraria Vista Florida, Instituto Nacional de Innovación Agraria (INIA), Lambayeque, Perú

**Keywords:** Dairy production, nutritional quality, native goats, dietary supplementation, milk quality

## Abstract

**Objectives:** In South America, goats largely depend on natural pastures, which often lack adequate nutrition. This innovative study ventured into uncharted areas by examining the potential of incorporating sweet potato vine flour (SPVF) into goat diets, specifically focusing on its effects on both the quantity and quality of milk produced.

**Materials and Methods:** A carefully monitored cohort of twelve female Creole goats, aged between 2 and 3 years, participated in a sophisticated completely randomized factorial design study. The research assessed varying levels of SPVF inclusion—spanning from the control group to significant incorporations of 2.5%, 5.0%, and 7.5%—observed meticulously over an eight-week period.

**Results:** The primary variables under detailed examination included daily milk yield, body weight, and milk quality. The latter underwent precise evaluation utilizing the advanced Milko Scan “FOSS”. Notably, the highest milk yield of 411 ± 7.92 ml/day was recorded at the 7.5% flour inclusion level, resulting in a statistically significant increase (*p* < 0.05) compared to the control group. Interestingly, an inclusion level of just 2.5% significantly enhanced the protein content to 3.84 ± 0.0159% and increased lactose levels to 4.54 ± 0.108%, with significant deviations in protein levels observed (*p* < 0.05).

**Conclusions:** SPVF improved milk production, but over 5% altered nutrition. Replacing corn with discarded sweet potato tubers is key for boosting milk production for small-scale goat farmers in Perú.

## 1. Introduction

The primary forage resource for small ruminants in South American countries comprises the native pastures prevalent across various ecosystems. However, these pastures frequently do not meet the nutritional requirements of the Creole goat [1, 2, 3]. The quality of available forage imposes limitations on the nutritional needs essential for growth, maintenance, reproduction, and production in goats, as evidenced by reduced voluntary intake and digestibility [4]. Presently, climate change exacerbates these challenges, hindering the fulfillment of nutritional needs and consequently impacting goat productivity, particularly in terms of milk production [5].

Goat milk is esteemed for its superior nutritional composition and functional attributes, rendering it a favored option among diverse consumer demographics, notably those with lactose intolerance or who seek natural dairy alternatives [6]. However, milk quality is highly dependent on the animals’ diet, highlighting the need for better feeding strategies to improve both productivity and the nutritional value of milk [7]. In this regard, the incorporation of agricultural by-products, such as sweet potato vine flour (SPVF), has received attention due to its accessibility, cost-effectiveness, and potential to augment the nutritional intake of ruminants [8, 9].

Sweet potato is extensively cultivated in developing nations [8]. This crop, traditionally grown for its tubers, results in the generation of crop residues such as leaves, stems, and root remains, which can be utilized as sources of fiber, proteins, and bioactive compounds in animal feed [10, 11]. At present, sweet potato crop residues are employed in the diet of goats on the northern coast of Peru. The production of dry matter (DM) per hectare in certain varieties of sweet potato ranges from 4.3 to 6.0 tons, with leaves and stems accounting for 64% of the fresh biomass. The crude protein content is approximately 11–17%, and its digestibility exceeds 62% [8, 12].

SPVF is distinguished by its content of digestible carbohydrates, dietary fiber, carotenoids, and antioxidants, all of which may exert a positive impact on the production and composition of goat milk. Additionally, its incorporation into the diet may enhance ruminal health, facilitate the synthesis of advantageous fatty acids, and augment the antioxidant profile of milk, which are essential factors in addressing current market requirements for healthier and more sustainable food products [13, 14].

However, the effect of different levels of SPVF inclusion on goat milk quality is not yet fully understood. It is imperative to ascertain the effects of this alternative feed source on parameters such as milk yield and composition (including fat, total solids, protein, and lactose); fatty acid profile; as well as antioxidant and sensory properties [15]. This study aims to assess the impact of diverse levels of SPVF inclusion in goat diets on milk production and quality, thereby providing scientific evidence to support its adoption as a viable and sustainable feed resource within goat production systems.

## 2. Materials and Methods

### 2.1. Ethical approval

The animal study protocol was approved by the Institutional Animal Care and Use Committee (IACUC) of the National Institute of Agrarian Innovation (INIA), Peru. The research was conducted in strict accordance with the principles outlined in the European Union Directive 2010/63/EU on the protection of animals used for scientific purposes.

### 2.2. Experimental site

The experiment was established during the arid season within the Goat Production Unit at the La Florida Experimental Station, part of the National Institute of Agrarian Innovation, in the district of Picsi, province of Ferreñafe, in the Lambayeque region of Peru (latitude 6°43′34″ south and longitude 79°46′49″).

### 2.3. Experimental design, feeding, and management

A total of twelve (12) local female Creole goats, aged 2 to 3 years and with an approximate weight of 35 ± 5 kg, were randomly allocated to a completely randomized design featuring a factorial arrangement. The goats were housed in individual pens within a common shed and were provided with Pennisetum, corn, and soybean concentrate as a forage base. The experimental treatments were structured according to the incremental inclusion of sweet potato vine flour (SPVF) in the diet: goats receiving a control diet with 0% sweet potato vine meal (T1), goats receiving a diet with 2.5% sweet potato vine meal (T2), goats receiving a diet with 5% sweet potato vine meal (T3), and goats receiving a diet with 7.5% sweet potato vine meal (T4).

### 2.4. Nutritional contribution of established diets

The moisture content, dry matter (DM), ether extract (EE), ash, crude protein (CP), crude fiber (CF), nitrogen-free extract (NFE), and caloric value of the diets obtained through a proximal chemical analysis are presented in Table 1. SPVF exhibited a concentration of 8.8% CP and 23.3% CF and a caloric value of 294.7 kcal, thereby contributing to the diet as an energetic fibrous food.

**Table 1.** Nutritional content of diets used for lactating native goats.

Subsistence allowance	Humidity %	US %	Ashes %	PC %	FC %	ELN %	Caloric value (kcal)
T1	12.1	8.4	23	13.6	7.6	56.1	359.5
T2	11.9	8.2	2.4	13.4	8.3	55.9	355.8
T3	11.8	8.1	2.6	13.3	8.8	55.5	352.9
T4	11.5	7.8	2.8	12.9	9.3	56.2	351.4
Sweet potato vine	3.6	2.2	2.6	8.8	23.3	59.6	294.7

### 2.5. Analysis of milk quality

The quantification of fat content, density, non-fat solids (total non-aqueous components, including fat, protein, lactose, and minerals), total solids (all components of milk except water and fat), protein, ash, lactose, pH, and acidity was conducted in the Milk and Meat Laboratory of the Faculty of Zootechnics at the National Agrarian University La Molina using the Milko Scan “FOSS” apparatus. This equipment’s calibrated milk analysis technology was specifically adapted for goats utilizing near-infrared spectrometry. For this analysis, a 100-cc sample of fresh goat milk was employed.

### 2.6. Statistical analysis

The response variables pertaining to milk quality were subjected to a completely randomized factorial ANOVA with week and treatment as factors. The treatments’ effect sizes were quantified using the Generalized Square Eta (GES) coefficient, which represents the proportion of total variance in the outcome variable that is attributable to that specific factor. Subsequently, means were compared utilizing Tukey’s post hoc test at a 0.05 significance level. To evaluate the temporal effects of the diets on milk production and body weight, a longitudinal analysis was conducted. The analyses were implemented using R version 4.1.1, employing the rstatix, emmeans, and ggplot libraries.

## 3. Results

### 3.1. Description of goat milk production and quality

Goats in the T4 group exhibited the highest milk production, yielding 411 ± 7.92 ml/day, whereas the T2 group demonstrated the lowest production at 308 ± 7.92 ml/day. Concurrently, the T4 group also recorded the highest body weight, measuring 45.5 ± 0.942 kg, in contrast to T1, which had the lowest body weight of 40.8 ± 0.943 kg (Table 2).

**Table 2.** Productive and quality variables of milk from goats fed with sweet potato vine flour.

Variables	T1	T2	T3	T4
Daily milk production (ml/day)	333 ± 7.92	308 ± 7.92	382 ± 7.92	411 ± 7.92
Body weight (kg)	40.8 ± 0.943	42.2 ± 0.942	46.7 ± 0.942	45.5 ± 0.942
Fat content (%)	5 ± 0.0811	4.71 ± 0.0811	4.87 ± 0.0881	4.65 ± 0.0774
Density (gm/ml)	1.0322 ± 0.0001	1.032 ± 0.0001	1.0321 ± 0.0002	1.0312 ± 0.0001
Non-fat solids (%)	10.02 ± 0.0677	9.84 ± 0.0677	9.92 ± 0.0736	9.61 ± 0.0647
Total solids (%)	14.4 ± 0.0709	14.1 ± 0.0709	14.2 ± 0.077	14.4 ± 0.0709
Protein (%)	3.81 ± 0.0159	3.84 ± 0.0159	3.79 ± 0.0173	3.72 ± 0.0152
Ash (%)	0.719 ± 0.008	0.694 ± 0.008	0.687 ± 0.0087	0.67 ± 0.0077
Lactose (%)	4.44 ± 0.108	4.54 ± 0.108	4.38 ± 0.118	4.41 ± 0.103
pH	6.54 ± 0.0322	6.45 ± 0.0322	6.51 ± 0.035	6.44 ± 0.0308
Acidity (%)	0.182 ± 0.0027	0.19 ± 0.0027	0.183 ± 0.0029	0.19 ± 0.0025

T1 exhibited a higher fat content at 5 ± 0.0811%, accompanied by non-fat solids at 10.02 ± 0.0677%, total solids at 14.4 ± 0.0709%, and ash at 0.719 ± 0.008%. Conversely, T2 demonstrated an elevated protein content of 3.84 ± 0.0159% and lactose levels of 4.54 ± 0.108%. Notably, T4 displayed heightened acidity, measured at 0.19 ± 0.0025% (Table 2).

### 3.2. Daily milk production and body weight

**Figure 1.**
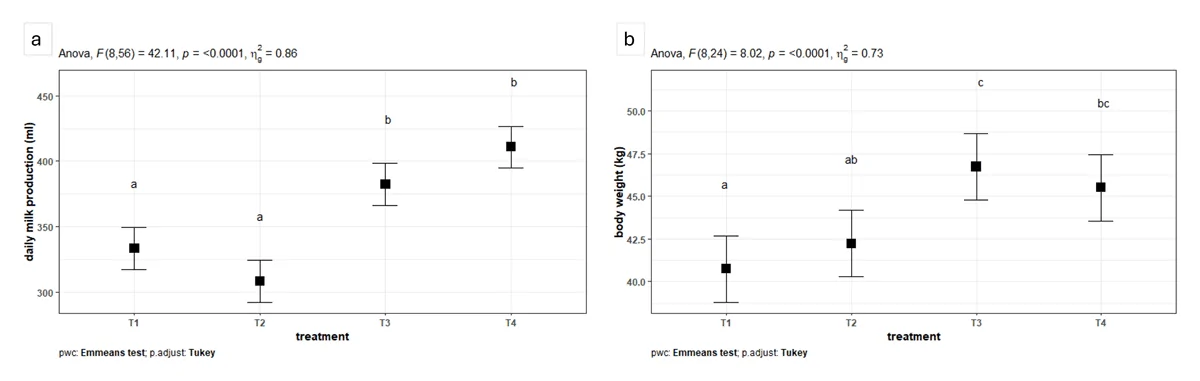
Effect of sweet potato vine flour inclusion on Creole goat productivity: (a) daily milk production and (b) body weight of lactating Creole goats fed diets with increasing levels of T1: 0%, T2: 2.5%, T3: 5%, and T4: 7.5%.

In Figure 1, it was observed that the incorporation of 2.5% SPVF (T2) did not exhibit statistically significant differences (*p* > 0.05) compared to the control group (T1) in terms of daily milk production and body weight. However, starting from a 5% inclusion level (T3), the lactogenic effect became apparent with statistically significant differences (*p* < 0.05). Conversely, the inclusion at the 7.5% level (T4) did not present statistically significant differences (*p* > 0.05) when compared to the T3 group.

### 3.3. Quality of goat milk

**Figure 2.**
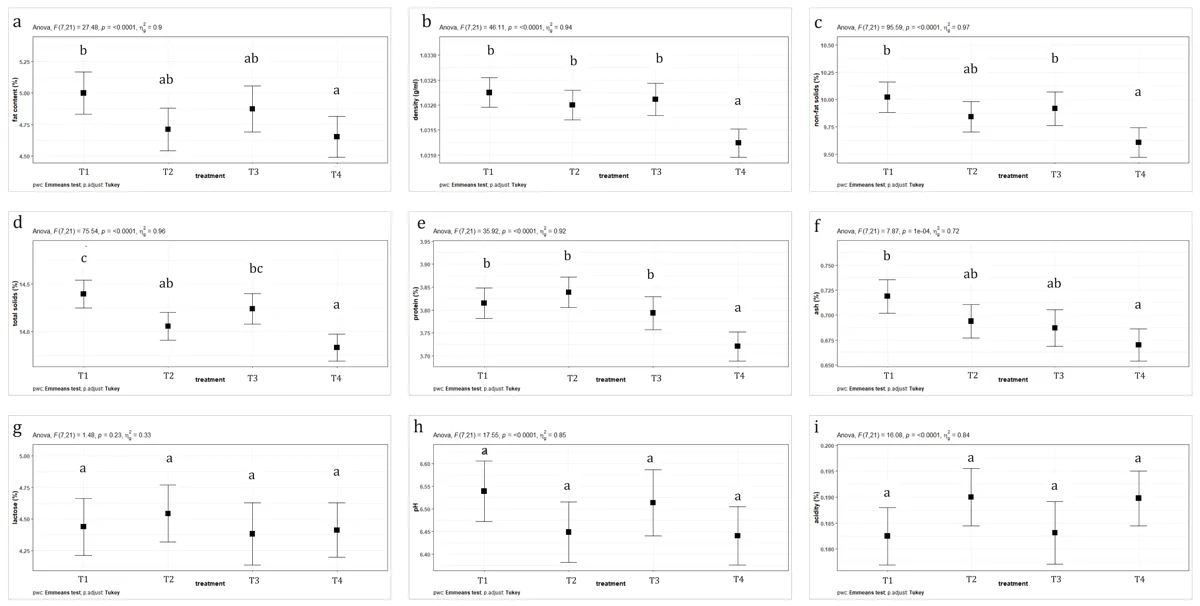
Milk composition of goats fed diets containing sweet potato vine flour: (a) fat, (b) density, (c) nonfat solids, (d) total solids, (e) protein, (f) ash, (g) lactose, (h) pH, and (i) acidity of lactating goats. Creole goats were fed diets with increasing levels of T1: 0%, T2: 2.5%, T3: 5%, and T4: 7.5%.

The fat and ash content of the control group (T1) exhibited statistically significant differences (*p* < 0.05) exclusively when compared with the group that contained 7.5% (T4). In contrast, comparisons with groups incorporating 2.5% (T2) and 5% (T3) revealed no statistically significant differences (*p* > 0.05). Furthermore, the groups with 2.5% (T2) and 5% (T3) displayed no differences when compared with the group containing 7.5% (T4) (*p* > 0.05) (Figures 2a, 2f).

No statistically significant differences in the density of milk and protein were observed between the control group (T1) and the experimental groups incorporating 2.5% (T2) and 5% (T3) of SPVF (*p* > 0.05). This may be ascribed to the notion that the critical threshold of excessive fiber consumption in the diet, which possesses the capacity to alter the equilibrium of ruminal microbiota, has yet to be exceeded. Conversely, significant differences were evident in the group containing 7.5% (T4) of SPVF (*p* < 0.05), with the control group (T1) displaying higher levels as depicted in Figures 2b and 2c, indicating that the critical threshold has been exceeded.

The content of non-fat solids in the control group (T1) did not exhibit significant differences (*p* > 0.05) when compared to the groups containing 2.5% (T2) and 5% (T3) of SPVF. In contrast, significant differences (*p* < 0.05) were observed with the group containing 7.5% (T4), with the control group (T1) demonstrating higher levels. Furthermore, the groups incorporating 2.5% (T2) and 7.5% (T4) also did not exhibit significant differences (*p* > 0.05) (Figure 2c).

Total solids in the control group (T1) had no differences (*p* > 0.05) from the group that included 5% (T3) of SPVF. Likewise, no differences were found between the groups that included 2.5% (T2) and 7.5% (T4) and the groups of 2.5% (T2) with 5% (T3). On the other hand, the differences (*p* < 0.05) were evident with the group that included 7.5% (T4) (Figure 2d), being greater in the control.

The data suggest that the control group (T1) exhibited an increased proportion of fat content, density, non-fat solids, total solids, protein, and ash, which might be attributed to its lower daily milk production [16]. Nonetheless, at certain levels of sweet potato flour inclusion, the evidence did not demonstrate the same clarity.

The analysis of pH, lactose content, and acidity indicated a lack of significant differences (*p* > 0.05) among the groups incorporating SPVF (Figures 2g, 2h, 2i). Consequently, it can be concluded that the inclusion of SPVF does not influence milk quality in terms of pH, acidity, and lactose content.

The statistical model explained nearly all the variance in daily milk production and body weight, as evidenced by η²G values approaching 1. This indicates a very strong effect of dietary treatments and a high degree of intraclass correlation within each group [17]. The large effect sizes (η²G) observed for milk production and body weight suggest a strong biological impact of the dietary treatments. However, these findings must be interpreted with caution due to the study’s small sample size, which reduces statistical power and the generalizability of the results.

SPVF costs $0.20/kg, less than corn, reducing feed costs (60–70% of production expenses). A 5% SPVF inclusion increases milk yield by 49 ml/day per goat, boosting income and covering SPVF costs. Sticking to 5% avoids reduced milk solids seen at 7.5%, maintaining milk quality and quantity.

## 4. Discussion

Creole goats from Latin America and the Caribbean exhibit a production range of 0.246–1.500 kg/day [18]. The mean production of the goats evaluated is more closely aligned with the herds in Loja, Ecuador, yielding 0.450 kg/day [19]; Oaxaca, Mexico, with 0.246 kg/day [20]; and Cochabamba, Bolivia, with 0.444 kg/day [21]. In Perú, research conducted by Ludeña and Bazan [22] in the town of Catacaos revealed a production level of 700 ml/day for Creole goats. The findings of our study suggest comparable production averages to those observed in goat herds from different nations, although the figures reported for Peru are marginally higher, which may be attributed to some level of crossbreeding involving the Peruvian Creole goat.

According to Gomez et al. [23], the body weight of female goats in the Apurímac region is reported to be 32 kg. In contrast, Palomino-Guerrera et al. [24] documented body weights ranging from 36.07 to 56.11 kg in herds from five locations in the Ayacucho region, both situated in the southern part of Peru. Conversely, in the Piura region to the north, Temoche et al. [25] reported body weights of 37.18 kg in female goats. The weight of the lactating goats in our study exceeded that from the Apurimac and Piura regions but remained within the range observed in the Ayacucho region. These discrepancies are likely attributed to environmental factors, including variations in feeding.

The administration of sweet potato vine silage to Nubian goats resulted in a significant increase in daily milk production (380 ml/day), indicating that the nutritional composition of sweet potato vine positively affects milk yield, in addition to enhancing body condition as evidenced by their body weight (28.45 kg) [26]. In our study, this effect was consistent in both milk production and body weight, with significant outcomes observed from the inclusion of 5% SPVF in the concentrate.

One-year-old Ethiopian Creole goats fed sweet potato vine at different levels (35% to 100%) with low-quality forage gained between 31.2 and 47.6 gm per day [27]. Similarly, in the diet of Ars-Bale goats, incorporating different proportions of sweet potato leaves into the forage base resulted in weight gains ranging from 20.83 to 60.13 gm per day, with optimal results observed at 25% and 50% leaf inclusion levels [28]. Sweet potato leaves are characterized by moderate concentrations of lysine (6.2 mg/kg DM), threonine (5.6 mg/kg DM), valine (7.2 mg/kg DM), isoleucine (5.7 mg/kg DM), methionine (2.6 mg/kg DM), and calcium (10.5 gm/kg DM) [29]. Therefore, the weight gain in our study’s goats was likely due to the high protein content of SPVF.

The protein, fat, and total solids in milk produced by Nubian goats remained unchanged when fed with sweet potato vine silage [26]. Similarly, the incorporation of sweet potato leaves into a supplement for dairy goats crossed with the Etawa breed did not alter the lactose composition, specific gravity, or mineral content of the milk [30]. In our study, differences in milk composition were not observed until SPVF was introduced at 5% in the diet; however, a 7.5% inclusion resulted in compositional changes.

In assessing the production cost of a silage comprising sweet potato vine and wheat bran as a supplement to Pennisetum Purpureum cv. In South Africa for Holstein cows, no statistically significant differences in production were observed, although numerical variations were present. Nevertheless, the production cost associated with sweet potato vine silage was reduced, attributable to a lower intake arising from its higher neutral detergent fiber content [31]. Although our study did not provide production cost indicators, it is noteworthy that the moderate inclusion level of 5% in the ration might contribute to the low indicators.

Our research investigates sweet potato by-products to boost milk production and body weight in ruminants. In Vietnam, adding 40% non-marketable sweet potato tubers to Boer goats’ diet improved dry matter intake, nutrient digestibility to 79.0%, and nitrogen retention to 13.7 gm/day, highlighting their potential as an energy source [32]. We used sweet potato vine flour, rich in crude fiber (23.3% CF) and low in non-structural carbohydrates, unlike starchy tubers. The study shows optimal inclusion levels differ; vines maintain rumen stability at 7.5%, and tubers boost energy and protein without fiber issues. Farmers are advised to use vines as fibrous additions (up to 5%) and tubers as grain substitutes (30–40%) for more efficient, sustainable, and profitable Creole goats.

SPVF benefits nutrition due to its effective ruminal degradation. Cultivars have high degradability for dry matter (43.67%) and crude protein (49.80%), enhancing milk yield by providing energy and nitrogen. These vines have higher CP content (14.71%) and degradability than ‘Danchina’ (13.74% CP) [33]. High degradability might reduce milk solids at 7.5% inclusion, suggesting excess SPVF can alter rumen fermentation, impacting milk fat and protein. Confirm that SPVF depends on both the inclusion level and cultivar. Farmers should consider vine quality and variety, not just quantity.

The use of SPVF presents a highly promising, sustainable, and scalable feed strategy for smallholder goat farmers. However, realizing its full potential requires further targeted research to fine-tune inclusion levels, a thorough understanding of its economic and nutritional impacts, and the development of standardized processing protocols.

## 5. Conclusions

The inclusion of sweet potato vine flour (SPVF) in the diet of Creole goats demonstrated a significant, positive effect on milk yield, with the highest production achieved at the 7.5% inclusion level. However, the relationship between SPVF and milk composition is not linear and involves a clear trade-off. While a low inclusion level (2.5%) showed potential to enhance specific components such as protein and lactose, higher inclusion levels (5% and 7.5%) led to a dilution effect or metabolic shift, resulting in statistically significant reductions in fat, protein, density, and solids content compared to the control. Therefore, the optimal inclusion level depends on the producer’s objective: maximizing volume (favoring 5–7.5% inclusion) or prioritizing milk richness and composition (favoring a lower inclusion rate ≤ 5%). The strategic use of SPVF, particularly when combined with discarded sweet potato tubers to replace a portion of conventional grains like corn, remains a promising strategy to reduce feed costs and improve the economic resilience of small-scale goat production in Peru. Future research should focus on optimizing the inclusion level to better balance this yield-composition trade-off. In the face of constraints such as sample size and study duration, the findings compellingly demonstrate that SPVF emerges as a remarkably viable and enduring feed resource. It is imperative that future research endeavors prioritize the validation of these promising results over extended timeframes. Moreover, conducting in-depth economic analyses and rigorously exploring the impacts on milk’s functional properties are essential steps to unlock the full potential of this extraordinary agricultural byproduct.

## Data Availability

The data presented in this study are available from the corresponding author upon reasonable request.

## List of abbreviations

ANOVA, analysis of variance; CF, crude fiber; CP, crude protein; DM, dry matter; EE, ether extract; INIA, National Institute of Agrarian Innovation; NFE, nitrogen-free extract; SPVF, sweet potato vine flour; gm/ml, gram per milliliter; gm, gram; kg, kilogram; kg/day, kilogram per day; mg/kg DM, milligram per kilogram dry matter; ml, milliliter; ml/day, milliliter per day.
